# Diagnostic Syllabus of the Inflammation Commonly Met with in the Uterus and Vagina

**Published:** 1876-10

**Authors:** J. H. Ethbridge

**Affiliations:** Professor of Therapeutics in Rush Medical College, one of the attending Gynæcologists at Central Free Dispensary


### Selections.

### DIAGNOSTIC SYLLABUS OF THE INFLAMMATION COMMONLY MET WITH IN THE UTERUS AND VAGINA.

PREPARED BY J. H. ETHBRIDGE, M.D.,
Professor of Therapeutics in Rush Medical College, one of the attending Gynæcologists at Central Free Dispensary.

The greatest difficulty a young gynæcologist has to encounter is correct diagnosis. Writers are justified in publishing anything that will give him assistance in determining what ails his patient. The following syllabus, prepared by the writer several years ago for individual use, is published in the hope that others may be benefitted. Familiarity with anatomy and pathology is absolutely indispensable to profiting by this tabulation. Mastering them thoroughly often enables the gynæcologist to know almost certainly what to expect from the general symptoms, and using the means of diagnosis is only confirmatory of his diagnostic suspicions. The usual means of diagnosis may be thus arranged to show at a glance what they reveal:

### MEANS OF DIAGNOSIS.

| GEN'L SYMPTOMS. | TOUCH. | SPECULUM. | PROBE. |
|---|---|---|---|
| 1. Pain: Locality and character. Amount.<br><br>2. Leucorrhœa. Character. Amount. Constancy.<br><br>3. Menstruation: Regularity. Amount. Pain. | Reveals:<br>1. Perviousness of vag. canal.<br>2. Location, size, density and tenderness of the cervix.<br>3. Os open or v. v., soft or v. v., smooth or rough, moist or dry, enlarged or elongated or atrophied.<br>4. Presence or absence of hardness or tumefaction of recto-vaginal or cysto-vaginal spaces; also nature of the same<br>5. State of ovaries and pelvic areolar tissue determined by lateral and upward pressure.<br><br>By Conjoined manipulation:<br>6. Volume, shape, sensitiveness of uterus, ovaries, broad ligaments and bladder.<br><br>By Rectal Touch, double and single:<br>7. Condition of post wall of uterus. | 1. Reveals color of m. m. of vaginal tract and cervix and condition of os.<br>2. Nature of leucorrhœa.<br>3. Declares atrophy or hypertrophy of cervix.<br>4. Reveals nature of mucus abrasions and "ulcerations." | 1. Reveals capacity of uterus.<br>2. Existence of foreign growths.<br>3. Shows deviations of course of canal, and differentiates them from tumors.<br>4. Indicates Endometritis. |

A glance at the syllabus will serve to show its design. In such a condensed arrangemen of symptoms, extreme accuracy and exhaustiveness are impossible. One or all four of these generally recognized uterine inflammations may be present, at one and the same time, in the same patient. Novices are devised to systematically use the indicated "means of diagnosis," and habits of accurately observing will soon be engendered.

FOUR VARIETIES OF UTERINE

| MEANS OF DIAGNOSIS. | 1. METRITIS. | | 2. CERVICITIS. | |
|---|---|---|---|---|
| | Acute, *Very rare.* | Chronic. | Acute. | Chronic. |
| 1. Gen. sym | *General Symptoms:*<br>*a.* Violent pelvic pain, accompanied with rectal vesical and uterine tenesmus, and sometimes with nausea and vomiting.<br>*b.* Pressure over abdomen reveals great sensitiveness. | *General Symptoms:*<br>*a.* Dull, heavy, dragging pain in pelvis, increased by locomotion.<br>*b.* Defecation and coition painful.<br>*c.* Menses accompanied with pain, which begins several days previous.<br>*d.* Pain in mammæ during and before menstruation.<br>*e.* Darkening of areolæ of the breast<br>*f.* Nausea and vomiting.<br>*g.* Great nervous disturbance.<br>*h.* Pressure on rectum with hæmorrhoids and tenesmus.<br>*i.* Pressure on bladder with vesical tenesums. | See Acute Metritis. | *General Symptoms:*<br>*a.* Pain in back and loins.<br>*b* Pressure on bladder or rectum.<br>*c.* Painful and sometimes profuse menstruation.<br>*d.* Difficulty of locomotion.<br>*e.* Nervous disor ders.<br>*f.* Pain during sexual intercourse.<br>*g.* Dyspepsia, headache, general lastitude and debility. |
| 2. Touch. | *Touch:*<br>*a.* Vagina hot and dry, unless from co-existing endometritis there be purulent discharge.<br>*b.* Organ low in pelvis, os enlarged, cervix swollen, pressure on cervix very painful.<br>*c.* Painful tenderness most apparent upon rectal touch and conjoined manipulation. | *Touch:*<br>*a.* Enlargement<br>*b.* Tenderness. | | *Touch:*<br>*a.* Uterus low down.<br>*b.* Cervix large, swollen and painful and os may admit finger.<br>*c.* Usually tenderness. |
| 3. Specul'm | *Speculum:*<br>*a.* Usually produces too much pain to be used. | *Speculum:*<br>Nothing revealed specially. | | *Speculum:*<br>Confirms signs evinced by touch. |
| 4. Probe. | *Probe*<br>*a.* Produces intolerable pain and can not usually be resorted to. | *Probe:*<br>*a.* Usually reveals some flexion or version, tenderness. | | *Probe:*<br>Reveals great sensitiveness before reaching os internum, but nothin beyond that. |

### INFLAMMATION, DIVIDED AS FOLLOWS:

| 3. ENDOMETRITIS. | | 4. ENDOCERVICITIS. | |
|---|---|---|---|
| Acute. | Chronic. | Acute. | Chronic. |
| See Acute Endocervicitis. | *Gen. Symptoms:*<br>*a.* Leucorrhœa streaked, glairy and bloody.<br>*b.* Menstrual disorders..<br>*c.* Pain in back, groins and hypogastrium.<br>*d.* Nervous disorders.<br>*e.* Tympanitis.<br>*f.* Symptoms of pregnacy.<br>*g.* Sterility. | *General Symptoms:*<br>*a* Dragging weight and pain in pelvis, pain in back, groin and thighs.<br>*b.* Rectal and vesical tenesmus.<br>*c.* Purulent discharge sometimes bloody after three or four days.<br>*d.* Tympanitis and tender abdomen. | *General Symptoms:*<br>*a.* Dragging sensation in the pelvis.<br>*b.* Pain in back and loins increased by exercise.<br>*c.* Profuse irritating leucorrhœa, like boiled starch.<br>*d.* Menses too scanty or v. v., too frequent or v. v.<br>*e.* Nervous, irascible, moody or even hysterical.<br>*f.* Digestion impaired, ultimately spanæmia, sometimes nausea, etc. |
| | *Touch:*<br>*a.* Conjoined manipulation reveals tenderness of fundus. | *Touch:*<br>*a.* Vagina hot and dry or covered with above discharge.<br>*b.* Os gaping, cervix swollen and tender, body slightly enlarged, whole organ lower in pelvis than normal. | *Touch:*<br>*a.* Os in normal position, may be enlarged, lips puffy or may be roughened.<br>*b.* Pain results from placing the finger under the cervix and pressing upwards. |
| | *Speculum:*<br>*a.* Reveals nothing special. | *Speculum:*<br>*a.* Servix puffy, swollen and red. fluid exuding from os either clear, albuminous looking, mucopus or stringy and tenacious. | *Speculum:*<br>*a.* Long, stringy, tough, tenacious mucus, difficult to remove, exuding from os.<br>*b.* Cervix not usually enlarged, may be puffy and swollen and very red, as if ulcerated, due to removal of investing epithelium. |
| | ***Probe:***<br>*a.* Patulous os internum.<br>*b.* Uterine cavity prolonged.<br>*c.* Tenderness. Withdrawal followed by blood. | *Probe:*<br>*a.* Great tenderness throughout whole organ and removal followed by a few drops of blood. | *Probe:*<br>*a.* Meets with obstruction at os internum.<br>*b.* Does *not* produce pain by striking against the walls of the fundus, nor is its removal followed by blood or mucus. |

—*Chicago Medical Journal and Examiner.*

